# Supplementary material for: Infrared nano-spectroscopy of ferroelastic domain walls in hybrid improper ferroelectric Ca3Ti2O7
Source: Nat Commun. 2019 Nov 20;10:5235. doi: 10.1038/s41467-019-13066-9 (PMC6868197; doi:10.1038/s41467-019-13066-9)
Supplement: Supplementary file 1 — Supplementary Information [file 41467_2019_13066_MOESM1_ESM.pdf]

Supplementary Information for "Infrared nano-spectroscopy of ferroelastic domain walls in hybrid improper ferroelectric $Ca_3Ti_2O_7$"

K. A. Smith, et al.

Supplementary Note 1: Locating Domain Walls

Supplementary Figure 1 displays a photograph of a single crystal of $Ca_3Ti_2O_7$ in transmittance mode under crossed polarizers. The location of the ferroelastic domain walls is evident as a change in color. We also examine the quality of the surface under the microscope - searching for a smooth, flat area with a few clear domain walls. Once located, we check each area by high resolution atomic force microscopy as discussed in the text.

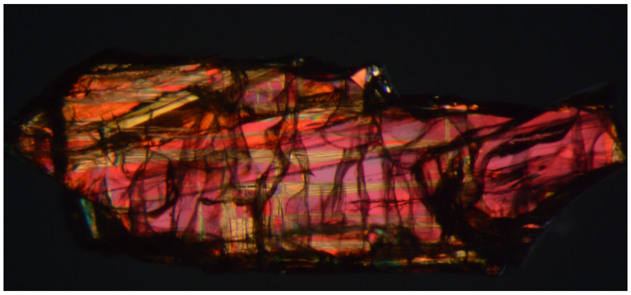

Supplementary Figure 1. Image of $Ca_3Ti_2O_7$ under crossed polarizers in transmittance mode. The direction of the polarizers are perpendicular in the plane of the image and are oriented to match the image edges.

Supplementary Table 1. Lattice parameters and symmetry adapted mode amplitudes for the bulk $A2_1am$ structure where (1) lattice parameters and atomic positions are relaxed with density functional theory (DFT), and (2) lattice parameters set to experimental values from Supplementary Reference [1] and atomic positions relaxed with DFT.

	(1) fully relaxed	(2) expt. latt. param.
a [Å]	5.3876	5.4172
b [Å]	5.4400	5.4234
c [Å]	19.3035	19.5169
$Q_{X_3^-}$ [Å]	1.202	1.147
$Q_{X_2^+}$ [Å]	0.868	0.894
$Q_{\Gamma_5^-}$ [Å]	0.598	0.557

Supplementary Note 2: Lattice-dynamical calculations

We employ density functional perturbation theory to calculate the phonon frequencies and eigenvectors of bulk $Ca_3Ti_2O_7$ in the $A2_1am$ space group. We consider two bulk $Ca_3Ti_2O_7$ structures with symmetry $A2_1am$ as starting points for our lattice-dynamical calculations: (1) a structure that is fully relaxed (lattice parameters and atomic positions) within density functional theory (DFT), and (2) a structure where the lattice parameters are fixed to the experimentally reported values [1] and only the atomic positions are relaxed. The lattice parameters and structural decomposition into symmetry adapted modes are reported in Supplementary Table 1 for both structures (1) and (2). Structure (1) was previously reported in Supplementary Reference [3] but is reproduced here for completeness. All numerical quantities reported in the main text were computed using structure (1). The calculated phonon frequencies and their symmetries are reported in Supplementary Table 2. Due to the large number of phonons present in the complex $Ca_3Ti_2O_7$ crystal structure, we are not able to make a complete assignment between the calculated and experimentally observed phonon frequencies.

Supplementary Note 3: Comparing the near and far field spectra

Supplementary Figure 2 displays the response of $Ca_3Ti_2O_7$ as a function of frequency. Differences in the near and far field spectra result from the near field technique probing a combination of the *ab*-plane and *c*-axis responses whereas the traditional measurement probes the plane perpendicular to the light. Other differences arise at low frequency due to the near field setup loss of sensitivity when approaching the low frequency cutoff. Regardless, the prominent features of the spectra appear in both the responses.

When comparing the measured spectrum to theory, it's important to remember that all of the infrared-allowed phonons of $Ca_3Ti_2O_7$ (19 A_1, 19 B_1, and 17 B_2) have frequencies, linewidths, and oscillator strengths that can be determined from first principles lattice dynamics calculations. These calculations are done for the material - not for a particular measurement technique like far field transmittance, reflectance, attenuated total reflectance, or near field infrared. Moreover, when we project out various order parameter contributions to the totally symmetric A_1 modes, these projections are characteristic of the modes of the material - again not a function of the measurement method. We use these calculations to

guide our thinking.

Supplementary Note 4: Projection of phonon eigenvectors

The bulk $Ca_3Ti_2O_7$ structure with symmetry $A2_1am$ can be expressed as $\mathbf{R}_{A2_1am} = \mathbf{R}_{I4/mmm} + \mathbf{u}$, where $\mathbf{R}$ is a vector containing the atomic positions of all the atoms, and $\mathbf{u}$ is the displacement vector. The distortion $\mathbf{u}$ can be decomposed into symmetry adapted modes that transform like the irreducible representations of $I4/mmm$:

$$\mathbf{u} = \sum_{i=1}^{4} A_{i\Gamma_1^+}\mathbf{e}_{i\Gamma_1^+} + \sum_{i=1}^{7} A_{i\Gamma_5^-}\mathbf{e}_{i\Gamma_5^-} + \sum_{i=1}^{6} A_{iX_3^-}\mathbf{e}_{iX_3^-} + \sum_{i=1}^{2} A_{iX_2^+}\mathbf{e}_{iX_2^+}. \quad (1)$$

Here $\mathbf{e}_{i\tau}$ is a symmetry-adapted mode that transforms like irreducible representation $\tau = \{\Gamma_1^+, \Gamma_5^-, X_3^-, X_2^+\}$ and i sums over the number of modes that transform like each irreducible representation τ. The coefficients are the overlaps $A_{i\tau} = \mathbf{u} \cdot \mathbf{e_{i\tau}}$, note that these are the symmetry adapted mode amplitudes that are reported in Supplementary Table 1. In an analogous manner, the A_1 phonons can be decomposed into the same basis of symmetry

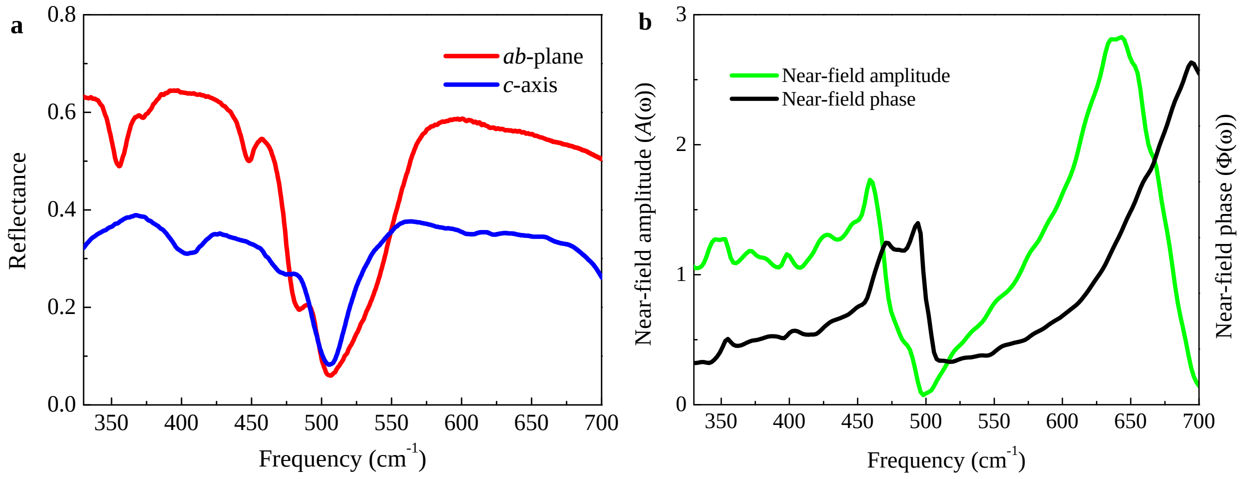

Supplementary Figure 2. Comparison of the far field and near-field spectra. (a) Polarization dependence of the far-field reflectance of $Ca_3Ti_2O_7$ at 300 K in the *ab*-plane and along the *c*-axis. (b) Near-field infrared amplitude and phase of $Ca_3Ti_2O_7$ at room temperature. The real and imaginary parts of a retarded Green's function are always related by a Kramers-Kronig relationship. The real part of the signal (the amplitude) therefore has an imaginary counterpart (the phase).

adapted modes:

$$\mathbf{e}_{A_1} = \sum_{i=1}^{4} A_{i\Gamma_1^+}\mathbf{e}_{i\Gamma_1^+} + \sum_{i=1}^{7} A_{i\Gamma_5^-}\mathbf{e}_{i\Gamma_5^-} + \sum_{i=1}^{6} A_{iX_3^-}\mathbf{e}_{iX_3^-} + \sum_{i=1}^{2} A_{iX_2^+}\mathbf{e}_{iX_2^+}. \tag{2}$$

The overlaps $A_{i\tau} = (\mathbf{e}_{A_1} \cdot \mathbf{e}_{i\tau})$ are reported in Fig. 4 (a) in the main text.

Supplementary Table 2. Experimental and calculated phonon frequencies for $\mathbf{Ca_3Ti_2O_7}$. The three right columns report the calculated phonon frequencies for structures (1) and (2) and their symmetry. The left three columns report the experimentally measured frequencies with Raman, infrared, and near field infrared (NFIR) spectroscopy.

<table>
<tr><th>Raman (cm^{-1})</th><th>Infrared (cm^{-1})</th><th>NFIR (cm^{-1})</th><th>Mode</th><th>Symmetry</th><th>Freq. (1) (cm^{-1})</th><th>Freq. (2) (cm^{-1})</th></tr>
<tr><td rowspan="2">783</td><td rowspan="2"></td><td rowspan="2"></td><td>1</td><td>A_2</td><td>789</td><td>780</td></tr>
<tr><td>2</td><td>B_1</td><td>775</td><td>769</td></tr>
<tr><td></td><td>721</td><td></td><td>3</td><td>B_2</td><td>707</td><td>668</td></tr>
<tr><td rowspan="2">676</td><td rowspan="2">626</td><td>660</td><td rowspan="2">4</td><td rowspan="2">A_1</td><td>652</td><td>637</td></tr>
<tr><td>610</td><td>652</td><td>637</td></tr>
<tr><td rowspan="3">550</td><td rowspan="3"></td><td rowspan="3">575
550</td><td>5</td><td>A_1</td><td>546</td><td>539</td></tr>
<tr><td>6</td><td>B_2</td><td>539</td><td>531</td></tr>
<tr><td>7</td><td>B_1</td><td>538</td><td>533</td></tr>
<tr><td rowspan="5">520</td><td rowspan="5">508</td><td rowspan="5"></td><td>8</td><td>A_2</td><td>522</td><td>517</td></tr>
<tr><td>9</td><td>A_1</td><td>517</td><td>508</td></tr>
<tr><td>10</td><td>B_1</td><td>515</td><td>498</td></tr>
<tr><td>11</td><td>A_2</td><td>512</td><td>494</td></tr>
<tr><td>12</td><td>B_2</td><td>507</td><td>494</td></tr>
<tr><td rowspan="4">488</td><td rowspan="4">483</td><td rowspan="4">475</td><td>13</td><td>B_2</td><td>499</td><td>490</td></tr>
<tr><td>14</td><td>A_1</td><td>495</td><td>486</td></tr>
<tr><td>15</td><td>B_1</td><td>473</td><td>469</td></tr>
<tr><td>16</td><td>B_1</td><td>470</td><td>460</td></tr>
<tr><td rowspan="2"></td><td rowspan="2"></td><td rowspan="2"></td><td>17</td><td>A_2</td><td>468</td><td>461</td></tr>
<tr><td>18</td><td>A_1</td><td>466</td><td>461</td></tr>
</table>

Continued on next page

Supplementary Table 2 – *Continued from previous page*

Raman (cm^{-1})	Infrared (cm^{-1})	NFIR (cm^{-1})	Mode	Symmetry	Freq. (1) (cm^{-1})	Freq. (2) (cm^{-1})
450	445	435	19	A_2	447	433
			20	B_2	441	438
			21	B_1	437	430
			22	A_2	436	428
		420	23	A_1	428	424
			24	B_2	404	396
361	372	375	25	A_2	388	381
			26	B_1	373	361
	355		27	B_1	361	354
			28	B_2	352	337
			29	B_1	334	328
320	321		30	A_1	328	317
			31	A_2	326	320
			32	A_2	317	306
295	301		33	A_1	308	300
			34	B_1	307	298
			35	B_2	297	289
			36	A_1	293	284
			37	B_2	290	282
271	262		38	A_2	273	260
			39	B_2	272	259
245	243		40	B_1	268	262
			41	A_1	266	257
			42	B_2	249	242
			43	B_1	248	240
			44	A_1	246	240

Continued on next page

Supplementary Table 2 – *Continued from previous page*

Raman (cm^{-1})	Infrared (cm^{-1})	NFIR (cm^{-1})	Mode	Symmetry	Freq. (1) (cm^{-1})	Freq. (2) (cm^{-1})
			45	A_1	237	225
			46	A_2	234	225
			47	A_1	227	217
			48	A_2	225	215
			49	B_1	224	216
214	203		50	B_2	213	204
			51	B_1	212	202
			52	A_1	211	206
			53	A_2	205	197
			54	B_2	204	196
170	166		55	B_2	180	174
			56	A_2	179	170
			57	A_2	178	168
			58	B_1	174	165
			59	A_1	174	169
			60	B_1	170	164
	156		61	B_2	165	154
			62	B_1	161	153
			63	A_1	157	150
			64	A_2	139	133
			65	A_1	138	130
			66	B_1	131	117
	128		67	B_1	129	118
	111		68	A_1	109	108
			69	A_2	107	103

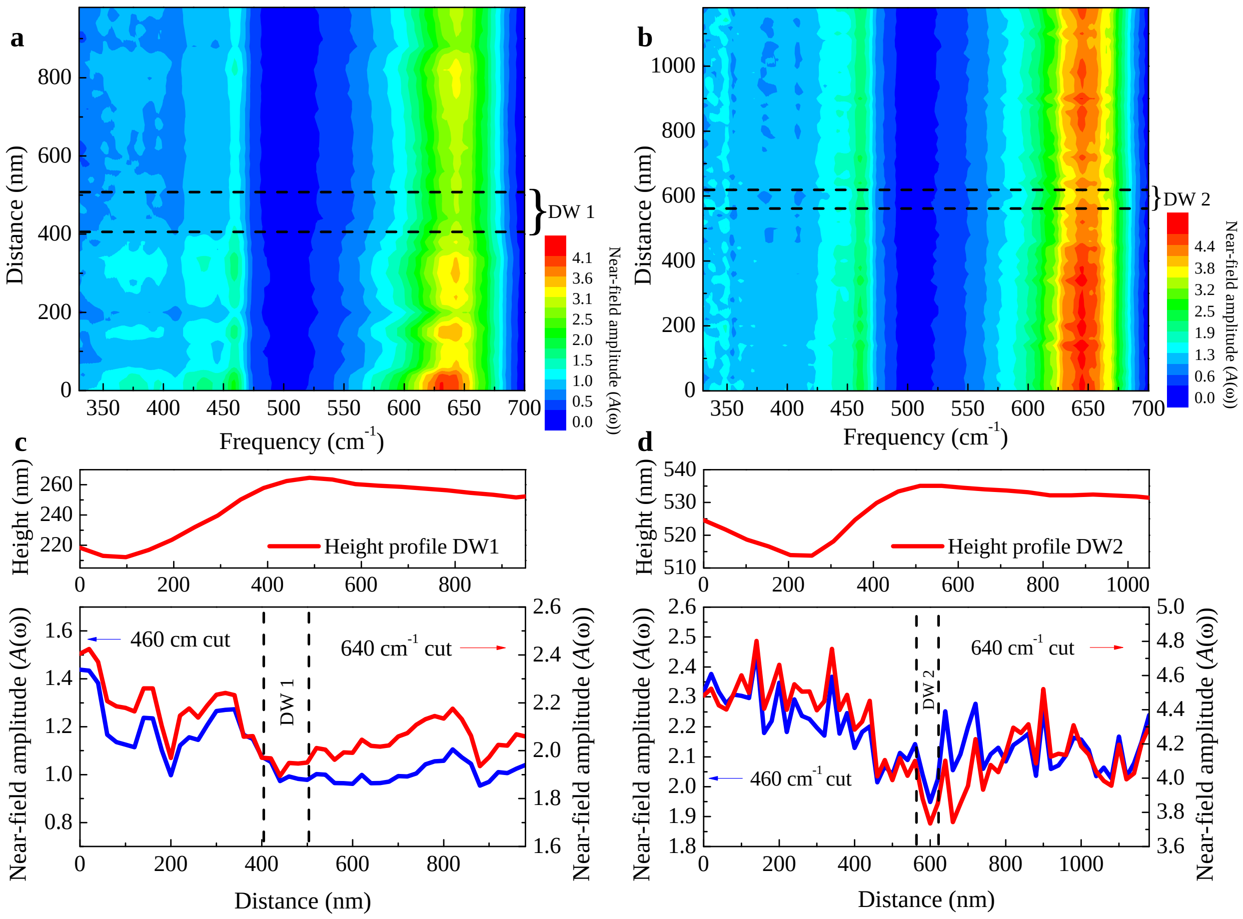

Supplementary Figure 3. Third harmonic response across DW 1 and 2. (a, b) Contour plot of the third harmonic near-field amplitude across DW 1 and 2. The color scales are slightly different due to the signal size. (c, d) Height profile (above) and fixed frequency cuts of the third harmonic near-field amplitude (below) as a function of distance across DW 1 and 2.

Supplementary Notes 5: Comparing second and third harmonic response

The synchrotron infrared nano spectroscopy setup enables simultaneous collection of higher harmonic data. Although the higher harmonic data is generally considered to have more sensitivity to the near-field, it also suffers from having lower signal-to-noise ratios. Prior work with this technique indicate that second harmonic detection is an appropriate compromise between the signal-to-noise and near-field sensitivity with good rejection of far-field artifacts as detailed by Supplementary Reference [4] in the paper. Within signal-to-noise limits, we generally find no (or very few) differences between the second and third harmonic signals

and thus believe the second harmonic channel to be mostly free from far-field artifacts. That said, we wanted to test this expectation with a direct comparison. Comparing contour plots in the main text with the data in Supplementary Figure 3 above, one can see that there is an excellent match between second and third harmonic signals across a ferroelastic domain wall.

Supplementary Note 6: Scanning multiple ferroelastic domain walls

As part of this work, we evaluated the nano-spectroscopic response of a number of domain walls. All together, we examined approximately 12 walls. Due to the complexity of our experimental technique, we were not able to measure a sufficient number of domain walls to compute quantities statistically averaged over many domain walls. The domain walls that we imaged have many similar characteristics, but they also have variations, which is why we choose to show two examples (DW 1 and DW 2) in the main text.

Supplementary Note 7: Locating and identifying ferroelectric walls

180° ferroelectric domain walls are challenging to locate because they meander between twin boundaries and are not readily apparent in AFM topography. Locating them a multi-step process. We first take a high resolution AFM image of a large, flat area of the crystal and use the position of at least two natural defects or step edges along with intimate knowledge of the relative x-y coordinates to denote position [Fig. 2(c), main text]. We then scan the same area with piezo-force microscopy to locate the ferroelectric domains and walls [Fig. 2(d), main text]. Overlaying the images reveals the location of the ferroelectric walls, which can then be re-located in the AFM field of view. By so doing, we can precisely locate ferroelectric domain walls in $Ca_3Ti_2O_7$ and scan across them with the near-field infrared technique.

Supplementary Note 8: Near-field imaging of 180° ferroelectric domain walls

We also searched for infrared signatures of ferroelectric domain walls in $Ca_3Ti_2O_7$. Our primary motivation was to reveal how local lattice distortions of ungerade symmetry drive new properties at the wall. Recent theoretical and electron diffraction work indicating that ferroelectric walls in $Ca_3Ti_2O_7$ may be composed of two twin boundaries (such that the microscopic structural distortions help avoid polar divergence) provided additional motiva-

tion for this effort [5]. Unfortunately, the low frequency limit of the near-field setup (330 cm1) precludes following the behavior of the Ca-containing modes (which contribute most of the polarization amplitude) along with the octahedral rotations and tilts that are so central to the trilinear coupling mechanism [3, 6–8], although if needed, one could project the appropriate irreducible representations onto the higher frequency modes to gain some insight. We therefore decided to try a line scan across a ferroelectric wall. We identified candidate ferroelectric walls for analysis and near-field line scans using a combination of AFM, piezoforce microscopy, and a careful examination of the ridges and topography of the crystal surface as illustrated in Fig. 2(c, d). We were not, however, able to discern a unique infrared signature across any of the six or seven ferroelectric domain walls that we examined. This places clear constraints on the local lattice distortions at ferroelectric walls in $Ca_3Ti_2O_7$ and suggests that they are well localized – if not atomically thin – as previously supposed [5, 9, 10]. We estimate that the 180° ferroelectric walls in $Ca_3Ti_2O_7$ are no more than 5 nm thick. This observation reinforces our interpretation that the ferroelastic walls are thick because, if they are not, we would not expect a signal from them - by analogy to the ferroelectric walls described here. This finding raises the interesting question of how two relatively thick ferroelastic walls combine to form such a thin, Néel-like ferroelectric wall [5]. Is there a unique cancellation of the structural distortions? Or does the interplay between charge and structure mitigate the charge divergence [5] and reduce the natural width of the structural distortion? More detailed near-field infrared imaging combined with other local probe techniques and theoretical modeling may be able to differentiate between these and other models.

Supplementary References

[1] Elcombe, M. M., *et al.* Structure determinations for $Ca_3Ti_2O_7$, $Ca_4Ti_3O_{10}$, $Ca_{3.6}Sr_{.4}Ti_3O_{10}$ and a refinement of $Sr_3Ti_2O_7$. *Acta Cryst.* **47**, 305-314 (1991)

[2] Oh, Y. S., Luo, X, Huang, F. -T., Wang, Y., & Cheong, S. -W. Experimental demonstration of hybrid improper ferroelectricity and the presence of abundant charged walls in $(Ca,Sr)_3Ti_2O_7$ crystals. *Nat. Mater.* **14**, 407-413 (2015).

[3] Nowadnick, E. A. & Fennie, C. J., Domains and ferroelectric switching pathways in $Ca_3Ti_2O_7$ from first principles. *Phys. Rev. B.* **94**, 104105 (2016).

[4] Khatib, O., *et al.* Graphene-based platform for infrared near-field nanospectroscopy of water and biological materials in an aqueous environment. *ACS Nano.* **9**, 7968-7975 (2015).

[5] Lee, M. -H., *et al.* Hidden antipolar order parameter and entangled Néel-type charged domain walls in hybrid imprper ferroelectrics. *Phys. Rev. Lett.* **119**, 157601 (2017).

[6] Benedek, N. A., Rondinelli, J. M., Djani, H., Ghosez, P., & Lightfoot, P. Understanding ferroelectricity in layered perovskites: new ideas and insights from theory and experiments. *Dalton Trans.* **44**, 10544-10558 (2015).

[7] Harris, A. B. Symmetry analysis for the Ruddlesden-Popper systems $Ca_3Mn_2O_7$ and $Ca_3Ti_2O_7$. *Phys. Rev. B.* **84**, 064116 (2011).

[8] Benedek, N. A. & Fennie, C. J. Hybrid improper ferroelectricity: a mechanism for controllable polarization-magnetization coupling. *Phys. Rev. Lett.* **106**, 107204 (2011).

[9] Oh, Y. S., Luo, X, Huang, F. -T., Wang, Y., & Cheong, S. -W. Experimental demonstration of hybrid improper ferroelectricity and the presence of abundant charged walls in $(Ca,Sr)_3Ti_2O_7$ crystals. *Nat. Mater.* **14**, 407-413 (2015).

[10] Cao, W. & Barsch, G. R. Landau-Ginzburg model of interphase boundaries in improper ferroelastic Perovskites of D_{4h}^{18} symmetry, *Phys. Rev. B* **41**, 4334 (1990)
